# Adolescent short video addiction in China: unveiling key growth stages and driving factors behind behavioral patterns

**DOI:** 10.3389/fpsyg.2024.1509636

**Published:** 2024-12-11

**Authors:** Jiaxiang Guo, Ran Chai

**Affiliations:** ^1^Yellow River Conservancy Technical Institute, Kaifeng, China; ^2^Henan Agricultural University, Zhengzhou, China

**Keywords:** adolescents, short video addiction, primary influencing factors, academic progression, academic pressure, stage-specific patterns

## Abstract

The proliferation of short video apps has brought to the forefront the issue of adolescent addiction, a challenge that resonates across Chinese society. Despite growing attention, a comprehensive understanding of the factors propelling addiction at various adolescent stages and its impact on academic performance remains scarce. This study aims to fill this void by identifying key growth stages and crafting targeted intervention strategies. Our investigation engaged 1,896 Chinese students, averaging 15 years old, at pivotal educational junctures. Employing a mixed-method approach, we utilized interviews and surveys, enhanced by fixed effects models and instrumental variables, to discern patterns in short video addiction. The study revealed stage-specific catalysts for addiction: social identity in junior high, academic stress in senior high, and personality traits in university students. A concerning trend was the double and triple rate of severe addiction among senior high students compared to their junior high and university counterparts, respectively, with a peak of 52.7% mild addiction in university. Our predictive model provides a holistic perspective on the drivers of addiction. This groundbreaking analysis delineates the complex interplay of adolescent short video addiction in China, under-scoring its profound implications for academic progression in senior high. The findings under-score the urgent need for tailored interventions to counteract the adverse effects of addiction.

## 1 Introduction

Since the rapid expansion of short video applications in China beginning in 2018, there has been increasing concern and attention regarding adolescent addiction to these platforms. According to the 53rd Statistical Report on the Development of the Internet in China published by the China Internet Network Information Center (CNNIC), as of December 2023, the number of adolescent internet users in China reached 230 million, with an average daily usage time of 2.5 h spent on short videos among adolescents (Center, 2024). Additionally, the 5th National Survey on the Internet Usage of Minors released by the Central Committee of the Communist Youth League of China in December 2023 reported that approximately 54.1% of underage internet users frequently watch short videos (Committee, 2023). Short videos have permeated every aspect of adolescents' daily lives, and the phenomenon of adolescent short video addiction has raised significant societal concerns. Research on adolescent short video addiction is, therefore, imperative.

This study categorizes adolescents into three stages: early adolescence (13–15 years old), middle adolescence (16–18 years old), and late adolescence (19–25 years old), roughly corresponding to the stages from middle school to university. The study defines addiction levels based on daily usage time, with < 1 h per day categorized as non-addicted, 1–2 h as mildly addicted, 2–3 h as moderately addicted, and 3 h or more as severely addicted. Current research on adolescent short video addiction primarily focuses on three areas: first, the impact of short video addiction on adolescents; second, the causes of adolescent short video addiction; and third, strategies for addressing adolescent short video addiction.

Research on the impact of short video addiction on adolescents encompasses various aspects, including mental health, social behavior, and academic performance. In terms of mental health, studies by Gentile et al. (2011) have shown a significant association between excessive use of electronic media, including short videos, and psychological issues such as depression and anxiety in adolescents. Marino et al. (2018) further pointed out that the use of short videos leads to an increase in feelings of loneliness and a decrease in life satisfaction among adolescents. Regarding social behavior, Vannucci et al. (2017) found that the increased frequency of short video usage among adolescents correlates with heightened feelings of social isolation, and that excessive addiction to short videos further reduces their social interactions in real life. As for the impact on learning, Ye et al. (2022) demonstrated that short video addiction negatively affects adolescents' attention span, learning motivation, and academic performance. Scott and Woods (2019) noted that frequent use of short videos impairs adolescents' ability to concentrate on their studies, thereby affecting their academic performance. Xu et al. (2023) also pointed out that the use of short videos negatively impacts adolescents' academic performance, with addiction to short videos leading to sleep deprivation, which in turn reduces effective study time and results in declining grades (Hale and Guan, 2015; Jiang and Yoo, 2024). Montag and Diefenbach (2018)'s research explores the “self-presentation” behavior on short video platforms, suggesting that adolescents may gradually become addicted to the virtual world in pursuit of likes and attention, leading to behavioral addiction. Similarly, Anderson et al. (2017) found a close relationship between short video use and behavioral addiction. In terms of self-identity and sense of achievement, the research by Hawi and Samaha (2016) found that adolescents' self-presentation behavior on short video platforms may affect their self-identity and sense of accomplishment, leading to a decline in their achievement motivation in real life, which in turn reduces their sense of self-identity (Nathawat and Singh, 2021). Anderson and Jiang (2018)'s research revealed that prolonged viewing of short videos may have negative effects on adolescents' cognitive development, particularly in areas such as information processing and decision-making. The above studies indicate that adolescent short video addiction has negative effects in multiple areas, potentially leading to issues such as difficulty concentrating, declining academic performance, deteriorating vision, sleep deprivation, and the onset of anxiety and depression (Lissak, 2018; Bozzola et al., 2022; Chao et al., 2023; Ming et al., 2023; Xu et al., 2023; Zhu et al., 2024). These findings underscore the importance of investigating the causes of adolescent short video addiction. Only through in-depth research into the factors contributing to short video addiction at different stages of adolescence can effective solutions and coping strategies be developed.

Current research on the causes of short video addiction among adolescents primarily focuses on four aspects: physiological factors, psychological factors, social factors, and technological factors. Physiologically, short videos provide rapid visual and auditory stimulation, frequently triggering dopamine release, which generates feelings of pleasure and satisfaction in adolescents, ultimately leading to dependency (Goto and Grace, 2005; Hyman et al., 2006; Kyu and Cho, 2018). Psychologically, adolescents under academic and life stress are more prone to developing an addiction to short videos (Tu et al., 2023). This is because short videos can help adolescents regulate negative emotions caused by stress, serving as a means of escaping from real-life pressures (Kardefelt-Winther, 2014). Additionally, some adolescents use short videos as a platform to showcase themselves, gaining likes and attention from others, thereby satisfying their need for social recognition (Kardefelt-Winther, 2014). In terms of social factors, peer groups' habits and attitudes toward short video use significantly influence adolescents, often leading to collective addiction. The family environment also plays a crucial role; the absence of parental companionship and lack of proper guidance increase the risk of short video addiction among adolescents (Bandura, 1986; Livingstone and Helsper, 2008; Chao et al., 2023). Interpersonal relationships and attachment to online platforms have a significant impact on short video addiction as well (Zhang et al., 2019). Technological factors also contribute to adolescent addiction to short videos. Short video platforms utilize big data and artificial intelligence to personalize content recommendations based on users' interests and behaviors, enhancing user engagement (Chen et al., 2017; Lv et al., 2022; Qin et al., 2022). The content of short videos is generally highly entertaining and fast-paced, easily capturing adolescents' attention and encouraging prolonged viewing. While these studies explain the causes of short video addiction among adolescents from different perspectives, they lack analysis of the primary influencing factors at different stages of adolescence. Furthermore, research on how these factors change across different stages is also insufficient. Understanding the primary factors driving short video addiction at different stages of adolescence is essential for developing targeted strategies to address this issue. Exploring the patterns of short video addiction across various developmental stages will better equip us to prevent and mitigate the negative impacts of short video addiction on adolescents.

The current research on strategies to address adolescent addiction to short videos is relatively underdeveloped, with existing strategies typically focusing on three main approaches. First, providing high-quality cultural and spiritual products to redirect adolescents' interests and attention. Second, enhancing supervision by schools and families (Throuvala et al., 2019; Liu et al., 2022). Third, emphasizing multi-sectoral collaboration in society to develop anti-addiction models within short video software, aiming to collectively address the issue of adolescent short video addiction (Ting and Chen, 2020; Zhou, 2022). However, there have been no reports of successfully implemented intervention strategies targeting adolescent short video addiction to date. This may be due to a lack of strategies that are specifically tailored to the different developmental stages of adolescents.

In summary, current research on adolescent addiction to short videos primarily focuses on the impacts of such addiction, with a consensus that it negatively affects multiple aspects of adolescent development. There is a recognized need to explore effective strategies to mitigate these negative impacts. To formulate effective coping strategies, it is crucial to investigate the causes of short video addiction. Although there is a significant amount of research on the causes of adolescent short video addiction, it often lacks a segmented analysis across different developmental stages of adolescence. This gap makes it challenging to develop effective strategies for addressing short video addiction among adolescents. While some researchers have explored various coping strategies, the absence of studies on the primary influencing factors of short video addiction at different stages of adolescence and the lack of research on the changing patterns of this addiction across these stages hinder the development of targeted interventions. Consequently, the existing strategies are not sufficiently specific or effective in their implementation.

This study addresses this gap by analyzing the primary influencing factors of short video addiction at different stages of adolescence, identifying the key stages—middle school, high school, and university—where adolescents are most susceptible to addiction. Based on this analysis, the study explores the patterns of short video addiction across these stages and examines the impact of such addiction on academic progression during these crucial periods. This research lays the groundwork for developing effective intervention strategies tailored to the different stages of adolescent development. To the best of our knowledge, this is the first report to identify the primary influencing factors and stage-specific patterns of short video addiction among adolescents.

## 2 Materials and methods

### 2.1 Participants

The participants in this study were drawn from the northern Chinese cities of Kaifeng and Zhengzhou. A longitudinal survey was conducted from 2018 to 2024, involving a total of 1,896 students. The junior high school participants were all in the 8^th^ grade, totaling 616 students, with 321 males (52.1%) and 295 females (47.9%). The senior high school participants were 2^nd^-year students, totaling 512, with 263 males (51.4%) and 249 females (48.6%). The university participants included 366 1^st^-year students, with 193 males (52.7%) and 173 females (47.3%), as well as 402 2^nd^-year students, with 198 males (49.3%) and 204 females (50.7%).

This study adhered to the ethical standards outlined in the 1964 Declaration of Helsinki. All participants and their parents provided informed consent, and the survey was conducted anonymously using a questionnaire-based approach.

During the sample selection process, we focused on 8^th^-grade, 2^nd^-year high school, and 1^st^- and 2^nd^-year university students. We utilized baseline survey data that allowed for panel data analysis. At the school level, sample selection was performed to mitigate endogeneity. Following the sampling rules of the China Education Panel Sur-vey (CEPS), “If the survey grade in a sampled school has only one or two classes, all classes are sampled; if the survey grade has three or more classes, two classes are randomly selected.” In accordance with these rules, we retained schools with exactly two sample classes and only included those schools where the average scores of the two classes were identical. Since certain class-level attributes may also influence student performance, retaining schools with equal average class scores helps approximate the balance of variables affecting performance between the two classes, thereby reducing endogeneity.

In the selected classes, a voluntary participation survey was conducted with all students to minimize selection bias. A longitudinal survey approach was adopted for those students who volunteered, with data collection occurring once per academic semester. The survey started in 2018 for both middle and high school students. For the middle school stage, students were selected starting from Grade 8 and continued for 6 years. For the high school stage, students were selected starting from Grade 10 and continued for 4 years, with data collected after the students' college entrance examination, including their exam results and university admission status. The university phase of the survey began in 2020, with data collected for 4 years starting from the 2^nd^ year of university for selected students.

In the final sample, 51.4% of the students were male, and 48.6% were female. The average age of the students was 15 years, with a standard deviation of 0.751. At this age, there is a notable lack of research on the primary influencing factors of short video addiction. Our study aims to explore the primary influencing factors and stage-specific patterns of adolescent short video addiction in northern Chinese cities.

### 2.2 Development of the short video addiction scale

Based on our preliminary questionnaire survey and interviews regarding adolescent short video addiction, we identified the five most frequently mentioned influencing factors, which accounted for 95% of all factors identified. In combination with existing research on the factors contributing to short video addiction, the scale used in this study was structured around five primary dimensions: academic pressure, peer group identification (group identity), family influence, personality traits, and entertainment needs (Supplementary Table S1). Additionally, the scale included sections on basic demographic information and usage habits to collect background data. The items on the scale were designed based on a comprehensive literature review and expert opinions, utilizing a five-point Likert scale (1 indicating strong disagreement, 5 indicating strong agreement) for scoring.

Academic pressure is considered one of the significant factors influencing adolescent behavior. Research indicates that excessive academic burdens and pressure to perform can lead students to seek relaxation and escapism (Liu and Lu, 2012; Hosseinkhani et al., 2020). Short videos, as an easily accessible and entertaining medium, have become a common choice for students to alleviate academic pressure (Mu et al., 2022; Sun et al., 2024). Consequently, the scale developed for this study includes items related to academic pressure to assess its impact on short video addiction. Example of the project: I feel that I use watching short videos as a way to escape from academic stress.

Peer group identification has a significant impact on the behavior and psychology of individuals during adolescence (Newman and Newman, 1976; Kiesner et al., 2002; Newman et al., 2007). To gain peer recognition and a sense of belonging, adolescents often mimic and participate in the activities of their peers (Grinman, 2002). The social features and interactive functions of short video platforms make them tools for students to maintain and enhance peer group identification. Therefore, the scale used in this study includes a dimension on peer group identification to evaluate the social motivations behind students' short video viewing behaviors. Example of the project: My friends were all watching short videos, so I started watching them too.

Family influence refers to the significant impact of the family environment and parental behavior on adolescents' use of online media (Lauricella and Cingel, 2020; Lee et al., 2022). The short video viewing habits of family members, family communication patterns, and parental education styles all influence students' short video usage habits. The scale developed for this study includes a dimension on family influence to examine the role of family factors in short video addiction. Example of the project: My family often watches short videos, so I follow along and watch them too.

Personality traits are also key factors influencing individual behavior. Research has shown that high susceptibility, poor self-control, and sensation-seeking personality traits are closely associated with addictive behaviors (Munno et al., 2016; Zeighami et al., 2021). The scale used in this study includes a dimension on personality traits to explore the influence of students' personality characteristics on their short video usage behaviors. Example of the project: I find that I easily get addicted to short videos.

Entertainment needs are a primary motivation for media use. According to the Uses and Gratifications Theory, individuals use media to satisfy specific needs (Ruggiero, 2017). The diverse content and instant gratification provided by short videos attract a large adolescent audience (Meng and Leung, 2021). The scale developed for this study includes a dimension on entertainment needs to assess the extent to which students watch short videos to fulfill their entertainment needs. Example of the project: The main reason I watch short videos is that they are very interesting.

### 2.3 Scale testing and validation

#### 2.3.1 Assessment of content validity

To ensure the content validity of the scale, we invited three experts in educational psychology and adolescent research to review the scale. These experts assessed the relevance and importance of each item and provided suggestions for revisions. We calculated the Content Validity Ratio (CVR) to ensure that all items possessed high content validity. The CVR calculation is based on experts' evaluations of the relevance of each item, with CVR values ranging from 0.62 to 0.89. Specifically, the CVR for the academic stress dimension was 0.78, the CVR for the peer group identification dimension was 0.82, the CVR for the family influence dimension was 0.75, the CVR for the personality traits dimension was 0.85, and the CVR for the entertainment needs dimension was 0.68. All of these values exceed the standard threshold of 0.5, indicating that the items in our scale possess good content validity (Smid et al., 2020).

#### 2.3.2 Pilot study

The sample for the pilot study included university students from different academic years and disciplines. We randomly selected 500 students from three universities located in two northern cities to ensure diversity and representativeness of the sample. These students covered various academic fields, including science and engineering, humanities and social sciences, and business, to reflect potential differences in short video usage behavior across different academic backgrounds.

Two primary data collection methods were employed: surveys and semi-structured interviews. The survey included all the items from the scale, along with additional demographic questions such as academic year, major, and gender. The survey was designed in an online format and distributed to participants via email and social media platforms. We ensured the anonymity and voluntary participation of the survey to encourage honest and open feedback. The interviews aimed to collect participants' understanding and feedback on the survey items. We invited 60 students who participated in the survey to take part in one-on-one interviews. The interviews focused on exploring the clarity, relevance, and potential misunderstandings of the scale items. The interviews were recorded and transcribed for detailed content analysis.

#### 2.3.3 Exploratory factor analysis

The purpose of EFA is to explore the underlying structure of the items and determine which items cluster together to form factors. We used Principal Component Analysis (PCA) and applied Kaiser normalized orthogonal rotation to simplify the factor structure. Prior to conducting the EFA, we tested the correlation matrix of the items to ensure that the KMO and Bartlett's test of sphericity results were within an acceptable range, indicating that the data were suitable for factor analysis. The results of the EFA revealed five main factors, which aligned with our theoretical dimensions: academic stress, peer group identification, family influence, personality traits, and entertainment needs. The factor loadings for each item were >0.4, indicating a strong association with their corresponding dimensions (Table 1).

**Table 1:** Exploratory factor analysis (EFA) and confirmatory factor analysis (CFA).

**Item number**	**Item description**	**EFA factor loadings**	**CFA factor loadings**	**Dimension**
Q1	I often watch short videos due to academic stress.	0.75	0.79	Academic pressure
Q2	I watch short videos because my friends are watching them.	0.82	0.85	Peer group identification
Q3	My family often watches short videos, and I am influenced by them.	0.68	0.72	Family influence
Q4	I find myself easily addicted to short videos.	0.76	0.78	Personality traits
Q5	I watch short videos mainly because they are entertaining.	0.90	0.92	Entertainment needs
Q6	I often watch short videos because I don't know how to pass the time.	0.88	0.90	Entertainment needs
Q7	I watch short videos because they help me relieve academic stress.	0.70	0.74	Academic pressure
Q8	I often watch short videos due to their novel content.	0.84	0.86	Personality traits
Q9	I watch short videos to temporarily forget family problems.	0.64	0.67	Family influence
Q10	I often watch short videos because of the social interactions they offer.	0.80	0.83	Peer group identification

#### 2.3.4 Confirmatory factor analysis

To further validate the results of the EFA, we conducted a CFA. CFA uses maximum likelihood estimation to estimate model parameters and provides model fit indices to assess how well the model fits the actual data. We used the following indices to evaluate model fit: Chi-square/degree of freedom ratio (χ^2^/df), Comparative Fit Index (CFI), Tucker-Lewis Index (TLI), and Root Mean Square Error of Approximation (RMSEA). The CFA results supported the five-factor structure of the scale, with all CFA indices falling within acceptable ranges (χ^2^/df < 3, CFI > 0.9, TLI > 0.9, RMSEA < 0.06) (Table 1).

Through both EFA and CFA, we are confident that the items in the scale effectively measure the five key dimensions of short video addiction, providing a solid foundation for further analysis.

#### 2.3.5 Evaluation of the scale's validity and reliability

Convergent validity was assessed by calculating the Average Variance Extracted (AVE) for each factor, with all factors exhibiting an AVE >0.5, indicating good convergent validity (Smid et al., 2020). Discriminant validity was evaluated by comparing the correlations between each factor and the other factors, with results showing low inter-factor correlations (all < 0.85), demonstrating good discriminant validity.

Internal consistency was assessed using Cronbach's Alpha coefficients, with all dimensions showing Alpha values >0.7, indicating good internal consistency. Correlation analyses among the dimensions further supported the scale's structural validity.

To evaluate the predictive validity of the scale, we conducted a correlation analysis between the scale results and those of other related scales (e.g., Mental Health Scale). The results showed a significant correlation between the Short Video Addiction Scale and the Mental Health Scale (r = 0.45, *p* < 0.01), indicating good validity in predicting short video usage behavior and its effects. A retest was conducted 2 weeks later on a subset of the sample, yielding a retest correlation coefficient of 0.82, demonstrating high test-retest reliability.

The Adolescent Short Video Addiction Scale developed in this study has undergone rigorous validity and reliability testing. The results indicate that the scale possesses good content validity, face validity, construct validity, convergent validity, discriminant validity, structural validity, predictive validity, and test-retest reliability, making it an effective tool for studying high school students' short video addiction behaviors and their impacts.

### 2.4 Questionnaire survey and statistical analysis

In this study, we employed the aforementioned scale to conduct a survey on short video usage behaviors among a total of 1,530 students. Questionnaires were distributed across two randomly selected middle schools, two high schools, and two universities. All participants voluntarily completed the questionnaires after receiving full informed consent. The data collection process strictly adhered to principles of anonymity and confidentiality to ensure the privacy of the respondents.

The collected questionnaire data were analyzed using SPSS statistical software. Independent sample *t-*tests and Analysis of Variance (ANOVA) were employed to explore the primary influencing factors of short video addiction at different stages of adolescence. Finally, a regression analysis model was used to evaluate the combined effects of the five factors—academic pressure, peer group identification, family influence, personality traits, and entertainment needs—on adolescent short video addiction. This model also examined the changes in primary influencing factors across different stages. Additionally, a longitudinal survey was conducted to analyze the impact of adolescent short video addiction on academic progression. All statistical analyses were conducted using two-tailed tests, with the significance level set at *p* < 0.05.

### 2.5 Construction of a predictive model for adolescent short video addiction

To predict adolescent addiction to short videos, a multiple linear regression model was employed. The dependent variable (Y) represents the degree of short video addiction, while the independent variables (X) encompass five dimensions: social identity, academic stress, family influence, entertainment needs, and personality traits. Separate regression models were constructed for each stage. The model is expressed as follows:


$$\begin{array}{l} \text{Y}=\beta_{0}+\beta_{1}{\text{X}}_{1}+\beta_{2}{\text{X}}_{2}+\beta_{3}{\text{X}}_{3}+\beta_{4}{\text{X}}_{4}+\beta_{5}{\text{X}}_{5}+\beta_{6}{\text{X}}_{6}+\epsilon \end{array}$$

Here, Y denotes the level of short video addiction, and X1, X2, X3, X4, X5 represent social identity, academic stress, family influence, entertainment needs, and personality traits, respectively. β0 is the intercept, β1, β2, β3, β4, and β5 are the coefficients to be estimated, and ϵ is the error term. The model parameters were estimated using Ordinary Least Squares (OLS) method. The validity of the model was verified through statistical tests.

## 3 Results

### 3.1 Analysis of short video addiction across three adolescent stages

**Figure 1:**
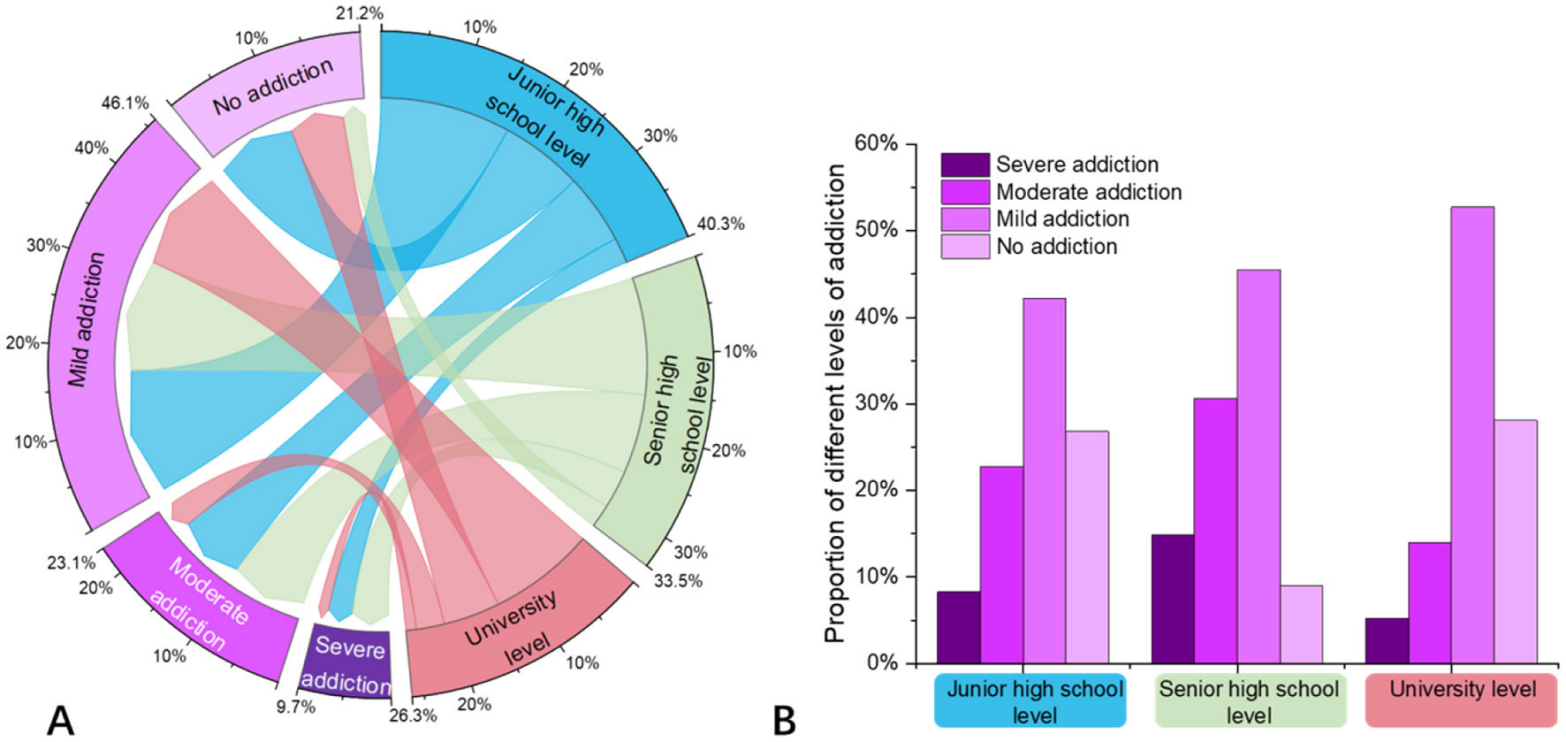
Analysis of short video addiction proportions across different adolescent stages. **(A)** Chord diagram analysis of short video addiction proportions across the three adolescent stages, and **(B)** bar chart analysis of short video addiction proportions across the three adolescent stages.

Through questionnaire surveys and scale analyses, it was found that approximately 22.7% of middle school students exhibited moderate short video addiction, with about 8.2% showing severe addiction. Among high school students, the rates of moderate and severe addiction were approximately 30.7% and 14.8%, respectively. For university students, around 13.9% demonstrated moderate addiction, while 5.3% were severely addicted. Notably, the proportion of severe addiction during the high school stage is nearly twice that of middle school students and three times that of university students, warranting significant attention. Overall, the level of addiction in the university stage showed a marked decline compared to middle and high school stages (Figure 1).

Overall, the probability of short video addiction is significantly higher during the high school stage compared to middle school and university stages. At the same time, the proportion of non-addicted individuals is highest during the university stage, at approximately 28.1%. The prevalence of mild addiction increases with age, with the highest proportion of mild addiction observed in the university stage, at around 52.7%.

### 3.2 Primary factors leading to short video addiction across three adolescent stages

**Figure 2:**
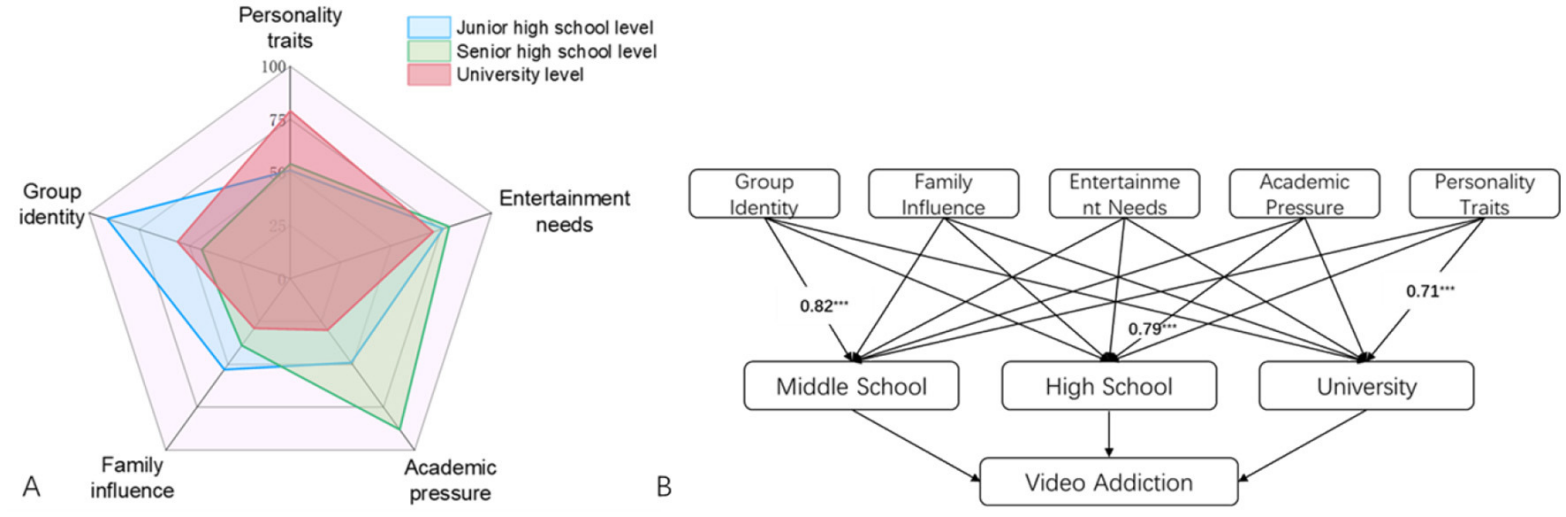
Primary influencing factors of short video addiction across different adolescent stages. **(A)** Primary influencing factors at different stages, **(B)** path coefficients of influencing factors, ***p < 0.001.

Data from adolescents with moderate or higher levels of addiction were selected from the survey and analyzed across five dimensions: personality traits, entertainment needs, academic pressure, family influence, and peer group identification. The results indicated that the primary influencing factor during the middle school stage is “peer group identification,” while “academic pressure” is the primary factor during high school, and “personality traits” is the primary factor during university (Table 2, Figure 2). This suggests that although there are common factors influencing adolescent short video addiction, there are distinct differences across stages. These differences should be carefully considered when developing prevention and intervention strategies.

**Table 2:** Factors influencing adolescent short video addiction.

**Stage**	**Variable**	**M**	**SD**	**Social identity**	**Academic stress**	**Family influence**	**Entertainment needs**	**Personality traits**
Middle school	Short video	1.81	0.62	0.82^***^	0.31	0.43	0.51^*^	0.23
High school	Short video	2.02	0.71	0.56^*^	0.79^***^	0.44	0.62^**^	0.32
University	Short video	1.54	0.53	0.33	0.43	0.34	0.65^**^	0.71^***^

^*^p < 0.05, ^**^p < 0.01, ^***^p < 0.001.

Construction of multiple linear regression models for predicting adolescent short video addiction across three stages:

(1) Middle School:

Y_J_ = −0.49 + 0.82X1 + 0.31X2 + 0.43X3 + 0.51X4 + 0.23X5–0.99

(2) High School:

Y_S_ = −0.71 + 0.56X1 + 0.79X2 + 0.44X3 + 0.62X4 + 0.32X5–0.90

(3) University:

Y_U_ = −0.92 + 0.33X1 + 0.43X2 + 0.34X3 + 0.65X4 + 0.71X5–0.96

The evaluation of model applicability indicates that the R^2^ values for models across all three stages exceed 0.8, suggesting that the models can significantly explain the variance in short video addiction. Moreover, the coefficients of the variables in all models are statistically significant, and their signs align with theoretical expectations, further substantiating the robustness of the models.

### 3.3 Changes in short video addiction across adolescent stages

**Figure 3:**
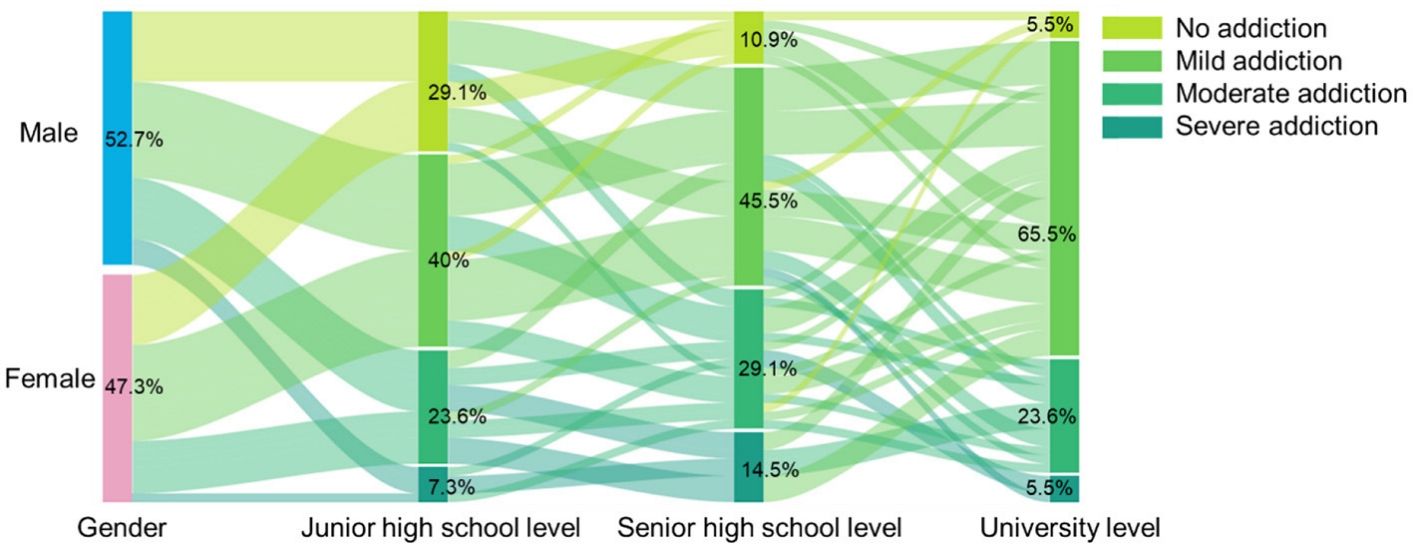
Changes in short video addiction across different adolescent stages.

Through a survey of 402 first-year university students, including semi-structured interviews, we analyzed their short video addiction at the middle and high school stages to understand the stage-wise changes in addiction. The results revealed a significant decrease in the proportion of students with moderate and severe addiction after entering university, while the proportion of students with mild addiction increased (Figure 3). This change may be related to the relatively more relaxed academic environment in university compared to high school, as well as the increase in extracurricular activities, which offer a wider range of entertainment options.

### 3.4 Impact of short video addiction on educational advancement

**Figure 4:**
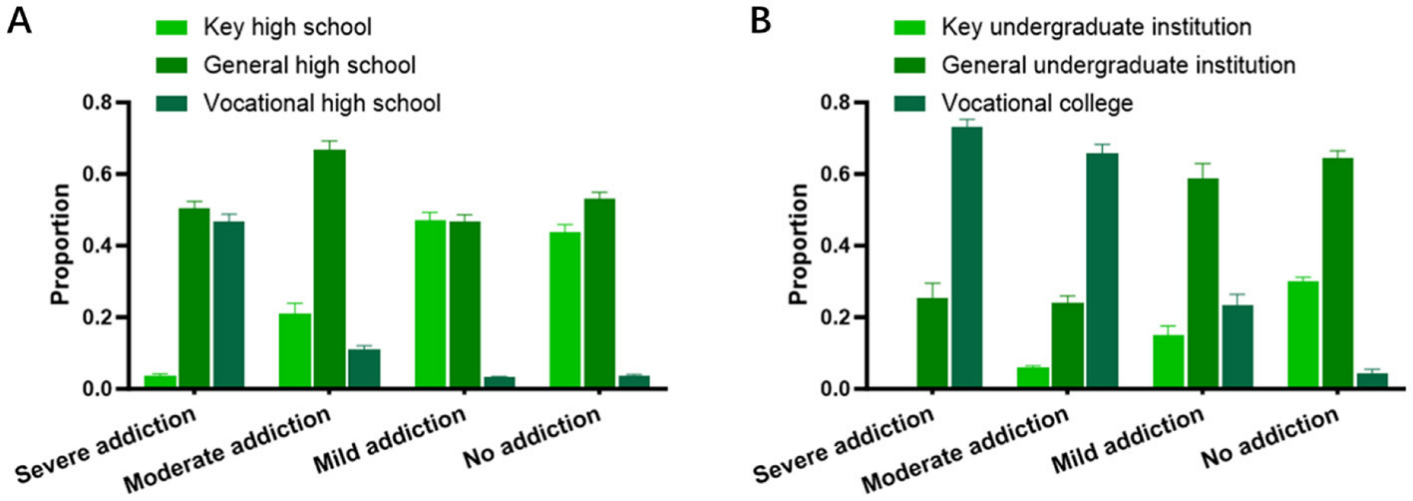
Impact of short video addiction on educational advancement. **(A)** Impact of short video addiction on middle school students' advancement to high school. **(B)** Impact of short video addiction on high school students' advancement to university.

Educational Advancement in this article refers to the process of entering from junior high school to high school, and the process of entering from high school to university. Based on a survey of 616 middle school students regarding their short video addiction, we tracked their educational advancement following the completion of middle school, specifically examining their success in gaining admission to key high schools, regular high schools, or vocational high schools. The results indicated that short video addiction does impact educational advancement at the middle school level. There was little difference between students with mild addiction and those without addiction; however, students with moderate and severe addiction were significantly less likely to be admitted to key high schools. Among students with severe addiction, only about 4% were admitted to key high schools, compared to more than 45% of students without addiction or with mild addiction (Figure 4A).

Similarly, in a survey of 512 high school students, we tracked their educational advancement following the completion of high school, focusing on their success in gaining admission to universities (based on college entrance examination scores). The results showed that none of the students with severe addiction were admitted to key universities, indicating that severe short video addiction has a more significant impact on college entrance exam outcomes for high school students. Among students with moderate addiction, only about 6% were admitted to universities, while approximately 30% of students without addiction were admitted to key universities—nearly double the rate of those with mild addiction. Furthermore, about 74% of students with severe addiction were only able to achieve scores sufficient for admission to vocational colleges (Figure 4B). This suggests that the impact of short video addiction on educational advancement is more pronounced during the high school stage compared to the middle school stage.

## 4 Discussion and conclusion

The detrimental effects of short video addiction on adolescents' growth and development have been widely recognized (Chao et al., 2023; Ming et al., 2023; Xu et al., 2023; Zhu et al., 2024). Researchers have explored the factors leading to adolescent short video addiction from various perspectives, including physiological, psychological, social, and technological factors (Bandura, 1986; Livingstone and Helsper, 2008; Kardefelt-Winther, 2014; Chen et al., 2017; Zhang et al., 2019; Tu et al., 2023). However, due to the rapid physical and mental development during adolescence and the ongoing changes in personality (Hazen et al., 2008; Orben et al., 2020), there are significant differences in cognitive maturity across different stages (Berenbaum et al., 2015; Dow-Edwards et al., 2019). Currently, there is a lack of research focusing on the primary factors influencing short video addiction at different developmental stages, which significantly hampers the formulation of effective prevention and intervention strategies for short video addiction. This study found that the primary factors influencing short video addiction change markedly across the three critical stages of adolescence—middle school, high school, and university. During the middle school stage, peer group identification emerged as the primary factor influencing short video addiction. In the high school stage, academic pressure became the leading factor contributing to short video addiction, while in the university stage, personality traits were the primary influencing factor.

These stage-specific changes may be related to the relatively young age of middle school students, who are at a critical period for self-identity and social identity formation (Pérez-Torres, 2024). Adolescents in middle school are in the process of seeking self-positioning and are likely to establish their identity through interactions and comparisons with peers. Peer relationships significantly influence adolescent behaviors and attitudes. Peer pressure and imitation are key drivers of short video addiction among middle school students. If the majority of the peer group is addicted to short videos, individuals may be inclined to imitate this behavior to integrate into the group and avoid marginalization or rejection (Nesi et al., 2018; Odgers and Jensen, 2020). In contrast, during the high school stage, academic pressure increases significantly compared to middle school, particularly during the critical period of preparing for college entrance exams. Students face heavy academic workloads, frequent examinations, and uncertainties about the future. Short videos, as a form of quick entertainment, provide instant psychological relaxation and satisfaction in a short time (Yang et al., 2023). Some high school students may neglect their academic tasks while watching short videos, leading to delays in their academic progress, which further exacerbates academic pressure. This negative cycle makes students more reliant on short videos to relieve stress, creating a vicious circle. At the university stage, adolescents exhibit a high degree of individual variability, with each person having different personality traits, interests, and emotional needs (Costa et al., 2019). Certain personality traits, such as high anxiety, introversion, or novelty-seeking, may make individuals more prone to short video addiction. Impulsivity and self-discipline, key personality characteristics, may play a crucial role in short video addiction. University students with high impulsivity may find it difficult to resist the instant entertainment and stimulation provided by short videos, leading to difficulty in controlling the time spent on these activities. Students with poor self-discipline may struggle to balance academics, social life, and entertainment, resulting in excessive use of short videos, which can adversely affect their daily life and studies.

Given the significant differences in the primary factors influencing short video addiction across different stages, prevention and intervention strategies should be tailored to address these stage-specific factors. During the middle school stage, efforts can be made to establish positive peer groups, reducing the risk of addiction due to integration into negative peer circles. Schools can also provide psychological counseling to help students recognize their value, build self-confidence, and reduce the likelihood of seeking peer validation through short video addiction. For high school students, teaching relaxation techniques, such as meditation and deep breathing, can help them manage stress without relying on short videos. Encouraging participation in physical activities, artistic creation, or other beneficial extracurricular activities can offer alternative leisure options, reducing their dependence on short videos. At the university stage, workshops on self-discipline and time management can help students prioritize tasks and allocate time effectively. Personality assessments can also be offered to help students understand their traits and learn how to manage the negative impacts of traits such as high anxiety or impulsivity.

The progression of short video addiction in individuals across different stages of development is dynamic. This study found that some students who were addicted to short videos during middle and high school showed reduced addiction or no longer exhibited addictive behaviors in university, while others who had not shown addiction or had only mild addiction in middle and high school developed moderate or severe addiction in university. These phenomena may be attributed to the fact that during middle and high school, adolescents are in a critical period of self-identity development, making them highly susceptible to peer influence. Additionally, the academic pressure in high school is significant, which can contribute to short video addiction as a coping mechanism. As students mature, their self-awareness tends to increase, and the academic pressure in university is typically lower than in high school. As a result, they may become more conscious of the impact that short videos have on their lives and studies, leading them to reduce their usage. Furthermore, university students often encounter more diverse social circles compared to their middle and high school years (Karimi and Matous, 2018; Anil et al., 2024). Some students may develop new interests through participation in clubs, volunteer activities, or other extracurricular engagements, reducing their reliance on short videos. However, the university phase also presents students with more complex challenges, such as employment and career choices (Nabi, 2003; Paviotti, 2020), which might lead some to increase their use of short videos as a form of escape from uncertainty and anxiety, potentially leading to addiction. Additionally, the more flexible and irregular daily routines in university, as compared to the structured schedules of middle and high school, may cause some students to use short videos to fill time gaps, resulting in habitual dependence.

This study also revealed that short video addiction during middle and high school significantly impacts academic advancement, particularly in high school. Among the surveyed students, none of those with severe addiction were admitted to top-tier universities, and nearly 74% of students with severe addiction could only achieve scores sufficient for admission to vocational colleges, compared to only about 4% of non-addicted students. This phenomenon likely results from the substantial time that students unknowingly spend on short videos due to their addiction. This time consumption directly reduces the time available for studying, which is particularly detrimental for middle and high school students with weaker self-management skills. High school students, facing greater academic pressure and heavier workloads, are more adversely affected by the time occupied by short videos. The instant feedback and sense of accomplishment provided by short videos (e.g., likes and comments) may replace the sense of achievement that students typically derive from academic success, thereby diminishing their interest in and motivation for learning. For high school students, this substitution effect can be especially pronounced, as academic achievements often require sustained effort, whereas the instant gratification from short videos is more immediately rewarding.

This study has certain limitations. First, since the sample primarily comes from northern cities in China, the generalizability of the findings may be limited. Second, the study relies on self-reported data, which may be subject to biases such as social desirability bias and recall bias. Lastly, the research mainly focuses on the short-term effects of short video addiction, with limited exploration of its long-term consequences. Future studies could consider a broader geographic distribution, employ more diverse data collection methods, and examine the long-term effects of short video addiction.

## 5 Conclusions

In conclusion, adolescent short video addiction exhibits stage-specific characteristics, with different primary influencing factors at each stage. As adolescents age, their patterns of short video addiction may change, with both positive and negative outcomes. Short video addiction has a significant impact on academic advancement during adolescence, with particularly profound effects during high school. Therefore, it is essential to develop prevention and intervention strategies targeting adolescent short video addiction, tailored to the specific characteristics of each developmental stage.

## Data Availability

The original contributions presented in the study are included in the article/Supplementary material, further inquiries can be directed to the corresponding authors.
