# Supplementary material for: Adolescent short video addiction in China: unveiling key growth stages and driving factors behind behavioral patterns
Source: Front Psychol. 2024 Dec 11;15:1509636. doi: 10.3389/fpsyg.2024.1509636 (PMC11669308; doi:10.3389/fpsyg.2024.1509636)
Supplement: Supplementary file 1 [file Table_1.DOCX]

Supplementary Material

Table S1 Adolescent Short Video Addiction Scal

| **Scale Dimensions** | **Questions** | | **Options (1-5, with 1 indicating strong disagreement and 5 indicating strong agreement)** |
| --- | --- | --- | --- |
| **1.Basic Information** | Age: | | |
|  | Gender: Male/Female | | |
|  | Grade: | | |
|  | Daily short video viewing time (hours): Less than 1 hour / 1-2 hours / 2-3 hours / More than 3 hours | | |
| **2. Usage Habits** | When do you usually watch short videos? | Morning / Noon / Afternoon / Evening / Late Night | |
|  | Where do you usually watch short videos? | At home / At school / Other places | |
|  | How often do you watch short videos? | Multiple times a day / Once a day / Several times a week / Occasionally | |
| **3. Motivation and Reasons** | | | |
| **1.1Academic Pressure** | Do you watch short videos to relax because of academic pressure? | | 1-5 |
|  | Do you think watching short videos helps you reduce academic pressure? | | 1-5 |
|  | Do you watch short videos more frequently when you have a heavy academic workload? | | 1-5 |
| **1.2Peer Group Identification** | Do you watch short videos to fit into a social circle? | | 1-5 |
|  | Do you consider your friends' or classmates' preferences when watching short videos? | | 1-5 |
|  | Do you think watching short videos enhances your sense of belonging in your peer group? | | 1-5 |
| **1.3Family Influence** | Do your family members often watch short videos? | | 1-5 |
|  | Do you watch short videos because of the influence of your family members? | | 1-5 |
|  | Do you think the family environment influences your short video watching habits? | | 1-5 |
| **1.4Personality Traits** | Do you consider yourself someone who easily gets addicted to certain things? | | 1-5 |
|  | Do you find it difficult to manage your time? | | 1-5 |
|  | Do you enjoy seeking novelty and excitement? | | 1-5 |
| **1.5Entertainment Needs** | Do you watch short videos for entertainment? | | 1-5 |
|  | Do you find short video content more attractive than other forms of entertainment? | | 1-5 |
|  | Do you watch short videos for extended periods because of their entertainment value? | | 1-5 |
| **4. Impact and Consequences** | Do you think watching short videos affects your studies? | | 1-5 |
|  | Do you think watching short videos affects your sleep? | | 1-5 |
|  | Do you think watching short videos affects your social life? | | 1-5 |
| **5. Self-Control and Reflection** | Do you think you can control the time you spend watching short videos? | | 1-5 |
|  | Have you ever tried to reduce the time you spend watching short videos? | | 1-5 |
|  | Do you think your addiction to short videos is a problem? | | 1-5 |
| **6. Family and School Influence** | What is your family's attitude toward your short video watching habits? | | 1-5 |
|  | Does your school have any regulations or recommendations to limit short video watching? | | 1-5 |
|  | Are you supervised by parents or teachers to control your short video watching time? | | 1-5 |
| **7. Additional Comments** | What other reasons do you think contribute to your or your classmates' addiction to short videos? | | Open-ended |
| Please fill in: | | | |
